# Sarco(endo)plasmic Reticulum Ca-ATPase-2 Gene: Structure and Transcriptional Regulation of the Human Gene

**DOI:** 10.1100/tsw.2002.228

**Published:** 2002-05-29

**Authors:** Angel Zarain-Herzberg, Georgina Alvarez-Fernandez

**Affiliations:** Laboratorio de Biología Molecular, Departamento de Bioquímica, Facultad de Medicina, Universidad Nacional Autónoma de México, México DF. 04510, Mexico

**Keywords:** heart, sarcoplasmic, reticulum, SERCA2, transcription, promoter, human, gene, hypertrophy, structure, ATPase, calcium, splicing

## Abstract

The sarco(endo)plasmic reticulum Ca-ATPases (SERCAs) belong to a family of active calcium transport enzymes encoded by the SERCA1, 2, and 3 genes. In this study, we describe the complete structure of the human SERCA2 gene and its 5’ -regulatory region. The hSERCA2 gene is located in chromosome 12 position q24.1 in Contig NT_009770.8, spans 70 kb, and is organized in 21 exons intervened by 20 introns. The last two exons of the pre-mRNA produce by alternatively splicing the cardiac/slow-twitch muscle-specific SERCA2a isoform and the ubiquitous SERCA2b isoform. The sequence of the proximal 225-bp regulatory region of the SERCA2 genes is 80% G+C-rich and is conserved among human, rabbit, rat, and mouse species. It contains a TATA-like-box, an E-box/USF sequence, a CAAT-box, four Sp1 binding sites, and a thyroid hormone responsive element (TRE). There are two other conserved regulatory regions located between positions -410 to -661 bp and from -919 to -1410 bp. Among the DNA cis-elements present in these two regulatory regions there are potential binding sites for: GATA-4, -5, -6, Nkx-2.5/Csx, OTF-1, USF, MEF-2, SRF, PPAR/RXR, AP-2, and TREs. Upstream from position -1.5 kb, there is no significant homology among the SERCA2 genes cloned. In addition, the human gene has several repeated sequences mainly of the Alu and L2 type located upstream from position -1.7 kb, spanning in a continuous fashion for more than 40 kb. In this study, we report the cloning of 2.4 kb of 5’-regulatory region and demonstrate that the proximal promoter region is sufficient for expression in cardiac myocytes, and the region from -225 to -1232 bp contains regulatory DNA elements which down-regulate the expression of the SERCA2 gene in neonatal cardiomyocytes.

## INTRODUCTION

The sarcoplasmic reticulum (SR) $Ca^{2+}$ transport ATPase activity determines the rate of contraction and relaxation of cardiac, skeletal, and smooth muscle. Three types of sarco(endo)plasmic reticulum $Ca^{2+}$-ATPase genes (SERCA1, SERCA2, and SERCA3) have been cloned which encode at least seven isoforms[1,2,3,4,5]. The SERCA1 gene is expressed in fast-twitch skeletal muscle and encodes two isoforms that are developmentally regulated, named SERCA1a (adult) and SERCA1b (neonatal)[1,2]. The SERCA2 gene encodes two isoforms produced by alternative splicing (SERCA2a and SERCA2b) which are expressed in a tissue-specific fashion[3,4]. The SERCA2a isoform is predominantly expressed in cardiac slow-twitch skeletal muscle, although it is also expressed at lower levels in smooth muscle and nonmuscle tissues[4]. The SERCA2b isoform is expressed ubiquitously in most cell types, including smooth muscle and nonmuscle cells[4]. It is noteworthy that the level of SERCA2a mRNA and protein in cardiac muscle and slow-twitch skeletal muscle is 10- to 50-fold higher compared to smooth muscle and nonmuscle cells[4]. The SERCA3 gene encodes three isoforms and is expressed in many cell types but predominantly in epithelial and vascular endothelial cells[5].

In order to understand the transcriptional mechanisms that regulate the human SERCA2 gene expression in the normal and hypertrophic heart, the 5'-flanking region of rabbit, rat, human, and mouse SERCA2 genes have been cloned and some functional analyses of the regulatory regions have been reported[6,7,8,9]. Using a stable cell line containing a -254 bp promoter SERCA2/CAT fusion construct of the rabbit gene, it has been demonstrated that the proximal 5'-flanking region is sufficient to regulate the expression of the SERCA2 gene during myogenic differentiation of the mouse skeletal cell line $C_2C_{12}$[6]. The rat SERCA2 gene has three functional TREs within the first 500 bp of promoter region[7,10,11,12]. One binding site for the THRα1 receptor is located within the proximal promoter region (-72 to -284 bp) of the SERCA2 gene (TRE-3)[7,10,11,12]. Transient cotransfection studies have demonstrated transactivation by thyroid hormone receptor (TR) α-1 isoform of rat and rabbit SERCA2promoter/CAT constructs containing the proximal promoter region[10,11,12]. However, to date, the transcriptional mechanisms involved in SERCA2 tissue-specific gene expression during cell differentiation, development, and growth are not fully understood.

In this study, we have elucidated the complete genomic organization of the human SERCA2 gene, identified the precise location of exon/intron junctions, and mapped the gene to chromosome position 12q24.1 according to its Contig localization. We have cloned a 2.9-kb fragment containing –2.5 kb of the hSERCA2 promoter region and engineered several deletion constructs. Sequence analysis of the cloned region revealed the presence of various potential transcription factor response elements. Functional analysis of SERCA2/Luc constructs demonstrates that the proximal –259 bp of regulatory region is sufficient to confer strong transcriptional activity in cardiac myocytes. In contrast, constructs containing at least 1.2 kb of regulatory sequences showed three- to fivefold lower transcriptional activity, suggesting the presence of DNA cis-elements down-regulating the transcription of the gene in neonatal rat cardiomyocytes in culture.

## RESULTS AND DISCUSSION

### Structural Organization of the Human SERCA2 Gene

Search for the SERCA2 gene in the human genome using BLAST analysis software from NCBI of the human SERCA2a and 2b mRNA sequences revealed that the entire gene is located within a fragment of 122,605 bp-long in the PAC clone RPC 13-305I20. The nucleotide sequence of the

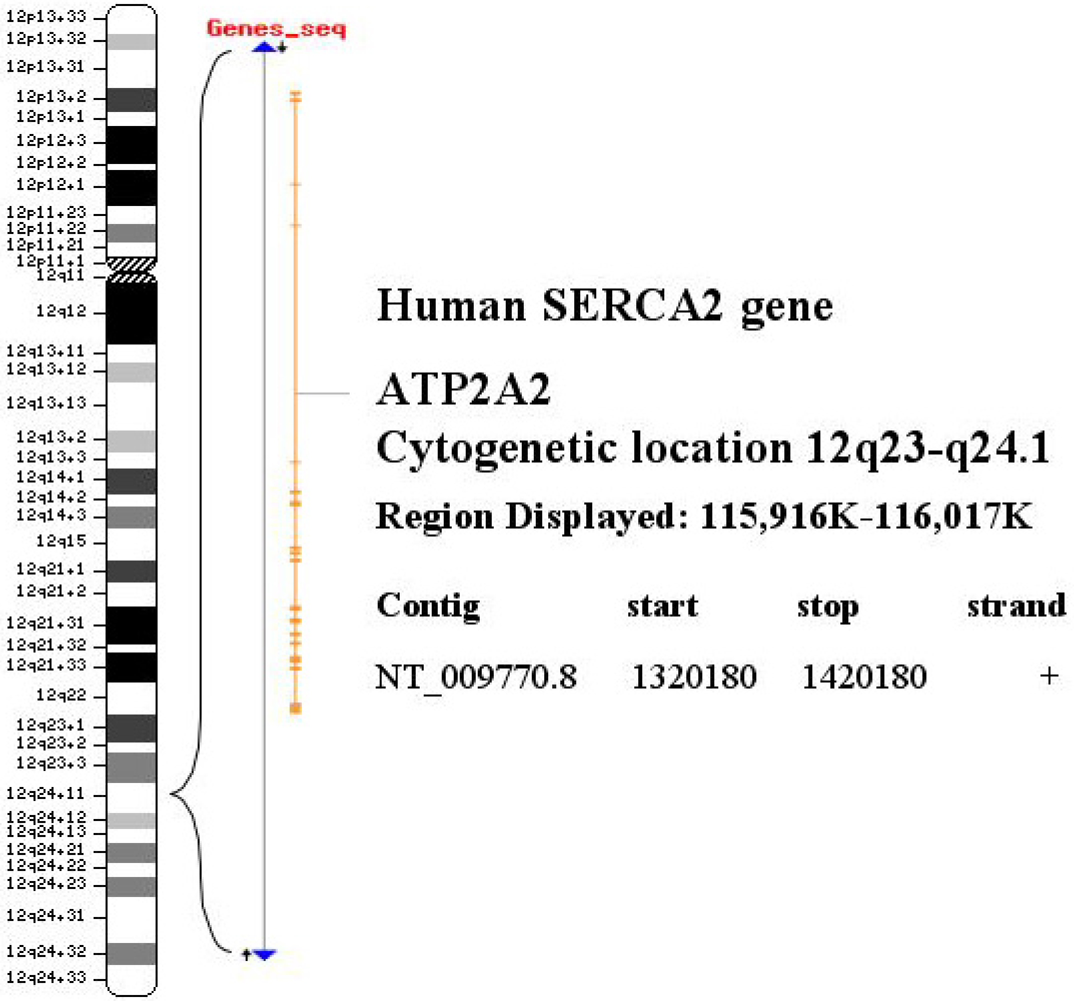

**FIGURE 1.** Chromosomal localization of the human SERCA 2 gene. The ideogram shows the localization of the SERCA2 gene in human chromosome 12. The schematic representation of exons of the SERCA2 gene in the 1320 to 1420 region of Contig NT_009770.8 in strand + is shown in gray.

clone was used to performed extensive nucleotide analysis to identify the precise location of exons within the genomic clone. The cytogenetic location of the SERCA2 gene is 12q23-q24.1. The SERCA2 gene is located between positions 115,916,977 and 116,017,977 bp of chromosome 12 in Contig NT_009770.8. This finding makes it possible to locate the SERCA2 gene more precisely to position 12q24.1 (Fig. 1). The genomic clone contains 41,909 bp of 5’-flanking sequence upstream of the transcription initiation site, including the promoter region. The transcribed genomic sequence of the hSERCA2 gene spans 70 kb, from position 41,910 to 111,737 of the genomic clone. The gene is organized in 21 exons intervened by 20 introns (Fig. 2). Introns 3,4,7,8,10,11,12,13,14,17, and 19 are in phase 0; introns 1,2,5,6,16, and 20 are in phase 1; and introns 9, 15, and 18 are in phase 2 (Table 1). Out of 20 introns, 19 contain the conserved GT and AG dinucleotides at the donor and acceptor sites, respectively, and only intron 12 starts with the GC sequence. The first exon is 646 bp long and contains 528 bp of noncoding sequence and 118 bp of coding sequence. The second exon is only 18 bp; it is in phase with exons 1 and 3. The longest intron is number 5 and is 26,255 bp long.

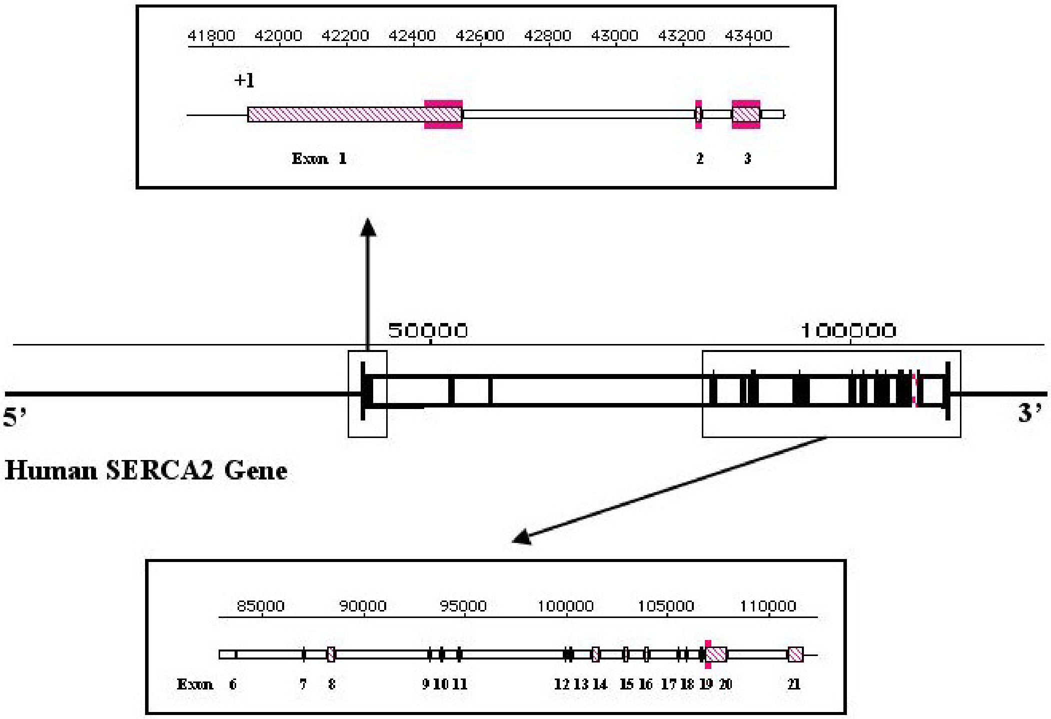

**FIGURE 2.** Genomic structure of the human SERCA2 gene. Schematic representation of the SERCA2 gene in clone RPC 13-305I20 is shown. The upper box shows the location of exons of the 5'-end region of the gene. Translated region is indicated with gray bars. The lower box indicates the position of exons 6 to 21.

## SERCA2a and SERCA2b mRNAs are Produced by Alternative Splicing

Alternative mRNA splicing is an essential process that regulates developmental stage and/or tissue-specific gene expression. This process can generate two or more mRNAs from a single pre-mRNA. Alternative splicing patterns can be classified as retained intron, selecting internal donor or acceptor sites, mutually exclusive exon, and cassette exon. For instance, the SERCA1 gene by a mechanism of mutually exclusive exon splicing produces two transcripts that encode two developmentally regulated isoforms expressed in fast-twitch skeletal muscle: the SERCA1a (adult isoform) and SERCA1b (neonatal isoform)[1,2]. Fig. 3A shows the location of the last three exons of the human SERCA2 gene. By a mechanism of alternative splicing, the last two exons of the SERCA2 pre-mRNA produce two different mRNAs that encode the SERCA2a and SERCA2b isoforms[4]. The SERCA2 pre-mRNA undergoes alternative splicing in a tissue- or cell-type–specific fashion, producing the SERCA2a transcript almost exclusively in cardiac and slow-twitch skeletal muscle, whereas the SERCA2b mRNA is present in a large variety of cells[4]. To produce the SERCA2b mRNA isoform, the pre-mRNA sequence of exon 20 is transcribed entirely. Exon 20 encodes the carboxyl terminal 89 amino acids of SERCA2b and contains at least 845 bp of 3'-nontranslated sequence. Exon 20 contains an internal intron donor site (GT) within its coding sequence immediately after the 40th amino acid that encodes (Fig. 3A). This donor site is preferentially used in cardiac and slow-twitch muscle by the splicing machinery to include exon 21 in the SERCA2a mRNA. As a result, the SERCA2a mRNA isoform lacks the sequence that encodes for the last 49 amino acids present in SERCA2b isoform, and instead it contains the 4 amino acids encoded by exon 21 (AILE), followed by a TAA stop codon and at

**TABLE 1**
**Exon and Intron Organization of the Human SERCA2 Gene**

| Exon | Exon Size bp | Exon 3' Sequence | 5' Junction | Intron | Intron Size kb | 3' Junction | Exon 5' Sequence | Spliced aa | Type |
|---|---|---|---|---|---|---|---|---|---|
| 1 | 121 | TCC AAC G | gt aggt | 1 | 0.7 | ctac ag | AG TTA CCG | Glu-40 | 1 |
| 2 | 18 | GAA GAA G | gt aatc | 2 | 0.09 | ttac ag | GA AAA ACC | Gly-46 | 1 |
| 3 | 83 | ATA TCT TTT | gt aagt | 3 | 9.22 | ttct ag | GTT TTG GCT | Val-74 | 0 |
| 4 | 105 | GTA TGG CAG | gt aagc | 4 | 4.47 | atac ag | GAA AGA AAT | Glu-109 | 0 |
| 5 | 139 | ATT GCT G | gt gagt | 5 | 26.25 | aatt ag | TT GGT GAC | Val-155 | 1 |
| 6 | 81 | CTC ACA G | gt aaat | 6 | 3.32 | gctt ag | GT GAA TCT | Gly-182 | 1 |
| 7 | 86 | CTG TTT TCT | gt aagt | 7 | 1.08 | tcct ag | GGT ACA AAC | Gly-211 | 0 |
| 8 | 465 | GTC TGC AGG | gt aaga | 8 | 4.58 | ctac ag | ATG TTC ATT | Met-366 | 0 |
| 9 | 89 | GGA GAA GT | gt gagt | 9 | 0.5 | gggc ag | G CAT AAA | Val-395 | 2 |
| 10 | 103 | TAC AAT GAG | gt aagt | 10 | 0.73 | atac ag | GCA AAG GGT | Ala-430 | 0 |
| 11 | 132 | TGC AAC TCA | gt gagt | 11 | 5.14 | ttgt ag | GTC ATT AAA | Val-474 | 0 |
| 12 | 124 | TTT GTG AAG | gc aagt | 12 | 0.10 | ttcc ag | GGT GCT CCT | Gly-515 | 0 |
| 13 | 219 | AAA TAT GAG | gt tagc | 13 | 0.94 | tttt ag | ACC AAT CTG | Thr-588 | 0 |
| 14 | 336 | ACA GCT ATG | gt gagc | 14 | 1.23 | tttc ag | ACT GGC GAT | Thr-700 | 0 |
| 15 | 221 | GTT GTC TG | gt aggt | 15 | 0.78 | aaac ag | T ATT TTC | Cys-773 | 2 |
| 16 | 203 | ATT GGC T | gt gagt | 16 | 1.45 | tccc ag | GT TAC GTC | Cys-841 | 1 |
| 17 | 86 | TAC CAG CTG | gt actc | 17 | 0.28 | ttgc ag | AGT CAT TTC | Ser-870 | 0 |
| 18 | 134 | CTC AAC AG | gt tagt | 18 | 0.62 | ctgc ag | C TTG TCC | Ser-914 | 2 |
| 19 | 118 | CCC TTG CCA | gt aagt | 19 | 0.08 | tgtc ag | CTC ATC TTC | Leu-954 | 0 |
| 20 | 121 | GAA CCT G | gt aaag | 20 | 3.97 | tttc ag | CA ATA CTG | Ala-994 | 1 |
| 20* | +1109 | CAT TTT AAA | | | | | | | |
| 21 | +798 | TTC TAT GAT | | | | | | | |

Exon sequences are indicated by uppercase letters and intron sequences by lowercase letters. Only the 3' end of exon 1 and the 3' ef exons 20* and 21 are shown. See text for details.

least 78 bp of 3'-nontranslated sequence. Therefore, if exon 20 is not spliced at the internal GT intron donor site, the mRNA produced is for the ubiquitous SERCA2b isoform. Although the mechanism involved for tissue-specific splicing of SERCA2 pre-mRNA is not fully understood, it is interesting to note that the 3' acceptor sites located in the 3'-region of intron 20 have consensus sequences for known intronic splicing suppressors (ISS) 5'-ACCUUGA-3', 5'-UUCUCU-3', and 5'-UUCCUU-3' (Fig. 3B)[13]. The same reversed 5'-AGUUCCA-3' element is also present in the alternatively spliced exon 9 of the F1 ATP synthase gamma subunit gene, and it has been shown to be essential for muscle-specific exon exclusion *in vivo*[13]. The 5'-region of SERCA2 exon 21 has a purine-rich sequence that matches exactly with the consensus sequence for an exonic splicing enhancer (ESE) element (5'-AAUGAAA-3') recently described for the F1 ATP synthase gamma subunit gene[14]. This putative ESE is located in the 5' region of exon 21 downstream from the putative ISS sequences located in the boundary of intron 20 with exon 21 (Fig. 3B), and may play a key role for splice site selection in alternative splicing to produce SERCA2a mRNA. Therefore, it could be speculated that the putative ESE present in exon 21 binds transacting regulatory factors present in cardiac and slow-twitch skeletal muscle, thus overcoming the influence of the suppressor elements present in intron 20 and favoring splicing that allows inclusion of exon 21 in the SERCA2a mRNA transcript. Regarding the identity of the transacting factors involved in muscle-specific splicing, it has been reported that the polypyrimidine tract-binding protein (PTB) is able to bind to ISS sequences similar to those present in the 3' flanking junction region of intron 20 of the hSERCA2 gene (Fig. 3B)[15]. Thus, it is possible that PTB functions as part of a constitutive exon selection mechanism. However, it does not seems to be the regulatory factor determining muscle-specific alternative splicing because its ubiquitous expression[15]. To date, the role of splicing factors such as hnRNP and other factors expressed in muscle tissue has to be examined for the SERCA2 splicing and experimental work is necessary to identify the proteins that bind these sequences.

**A**

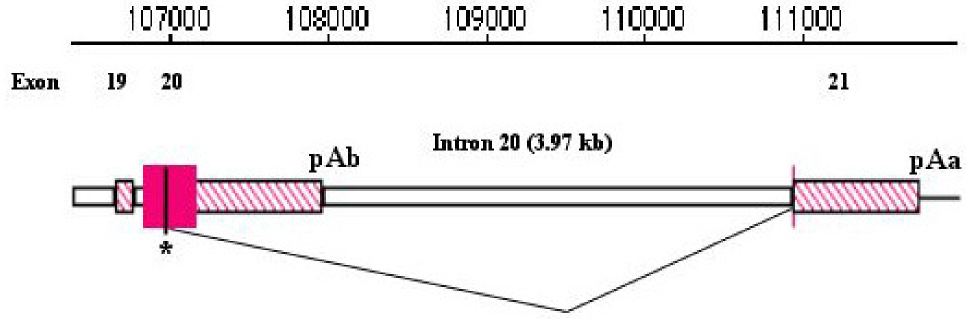

**B**

(110,752)5'GAUGCUACCCUGUGUGGGCGGCACCUCAGGGACAGUAAAUCAGAAAUGCUGGUCUU
GAAACCUUGAAAAGAUCAAGCUGAAUGUUCCUUUUCAUCUGUCGCUGUUGAUCUUCAUCUAUUUAA
AUAGGUAUUCUAACGUUUCCUCUCUGUAUUUCAUGAAGCUGAUUUCCUCUCUCUUUCCUUUUCAG/
**CAAUACUGGAGUAACCGCUUCCUAAACCAUUUUGCAGAAAUGUAAGGGUGUUCGGUUGCGUGCAUG**
**UGCGUUUUUAGCAACACAUCUACCAACCCUGUGCAUGACUGAUGUUGGGCAAAAAGAAAAGUAAAA**
**AACUUCCCAACUCACUUUGUGUU** 3' (111,093)

**FIGURE 3.** Splicing mechanism of the SERCA2 transcripts. (A) The structure of the 3'-end region of the gene is shown; the line indicates the splicing mechanism of the primary transcript to produce the alternate isoforms. The asterisk (*) indicates the position of internal donor site of splicing located in exon 20. pAa and pAb indicate the polyadenylation sites for the SERCA2a and SERCA2b mRNAs, respectively. (B) The nucleotide sequence of the junction region of intron 20 and exon 21 of the human SERCA2 pre-mRNA is shown. The consensus intronic splicing suppressor (ISS) sequences within intron 20 and the stop codon in exon 21 are underlined. The intron/exon junction acceptor site is indicated by a slash (/). The exon 21 sequences are in bold letters. The exonic splicing enhancer (ESE) consensus sequence is boxed.

## Sequence Analysis of the Regulatory Region of SERCA2 Gene

The 5'-regulatory regions of the mouse, rat, rabbit, and human SERCA2 genes have been cloned and sequenced[6,7,8,9]. Alignment analysis of the genomic DNA sequences of the 5'-flanking regions of these genes reveals important observations (Fig. 4). The first observation is that the 5'-regulatory sequence of the SERCA2 genes is organized in regions of homology containing cis-acting DNA regulatory elements. The first region includes 225 bp of proximal promoter region and contains a TATA-like-box (5'-GATAAA-3'), an E-box/USF consensus sequence (5'-CACATG-3'), a CAAT-box (5'-GCCAAT-3'), four consensus Sp1 binding sites (5'-GGGCGG-3' and 5′-CCGCCC-3'), and four 5'-GGGAGG-3' sequences that can also bind Sp1 and possibly other transcription factors, and a thyroid hormone responsive element (TRE) (5'-GGCCTCGATCCGGGTTC/ACTG/A-3'). The second region of homology is located between position -410 to -661 bp of the human gene and contains TRE-1, one serum response element (SRE), one muscle M-CAT, one activator protein-2 (AP-2), and one E-box/USF consensus sequences. Between position -662 to -992 bp there are only two short regions of homology, one between position -875 to -905 bp that contains an E-box element, another from position -944 to -975 bp that contains a T/A-rich sequence with homology to the muscle-specific SRE/CArG-box element. The third region of homology starts at position -919 and ends at position –1410, and contains one putative TRE, one E-box, and consensus M-CAT, MEF-2, N-FAT, OTF-1, and GATA elements. The second important observation is that upstream from this region there is no

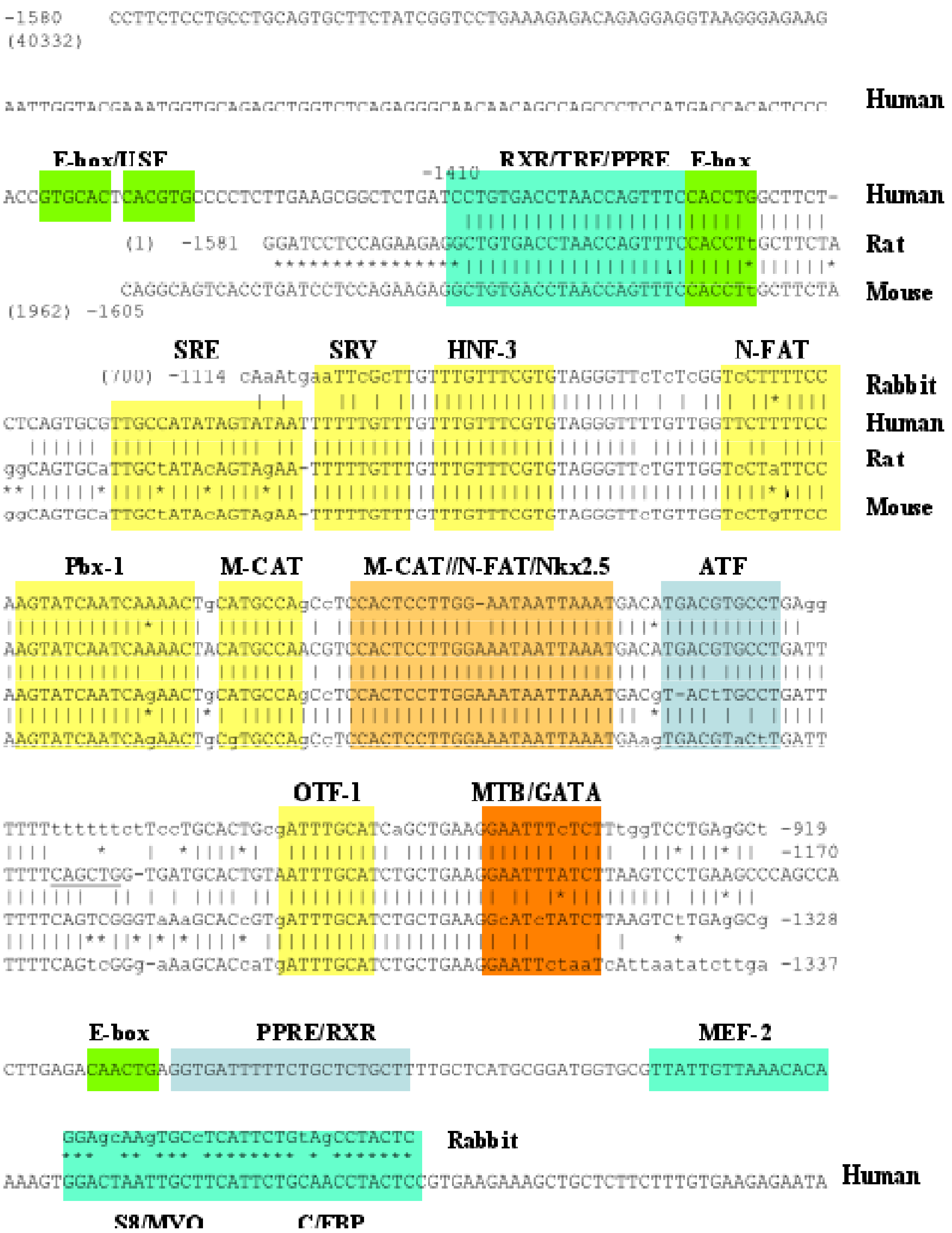

**FIGURE 4.** Nucleotide sequence alignment of SERCA2 gene 5'-regulatory regions. The identity in nucleotide sequence of 1.58 kb of SERCA2 regulatory sequences among human, rabbit, rat, and mouse species is indicated by a vertical line (|). An asterisk (*) indicates similarity only between two sequences. The consensus DNA sequences for various regulatory elements are shown in color areas, and ERSE is boxed. The 5'-nontranslated sequence of the first exon is in bold letters. MacVector 6.5.3, Blast NCBI, and Genomatix MatInspector Professional 5.1 were used to align sequences and to identify putative DNA binding motifs.

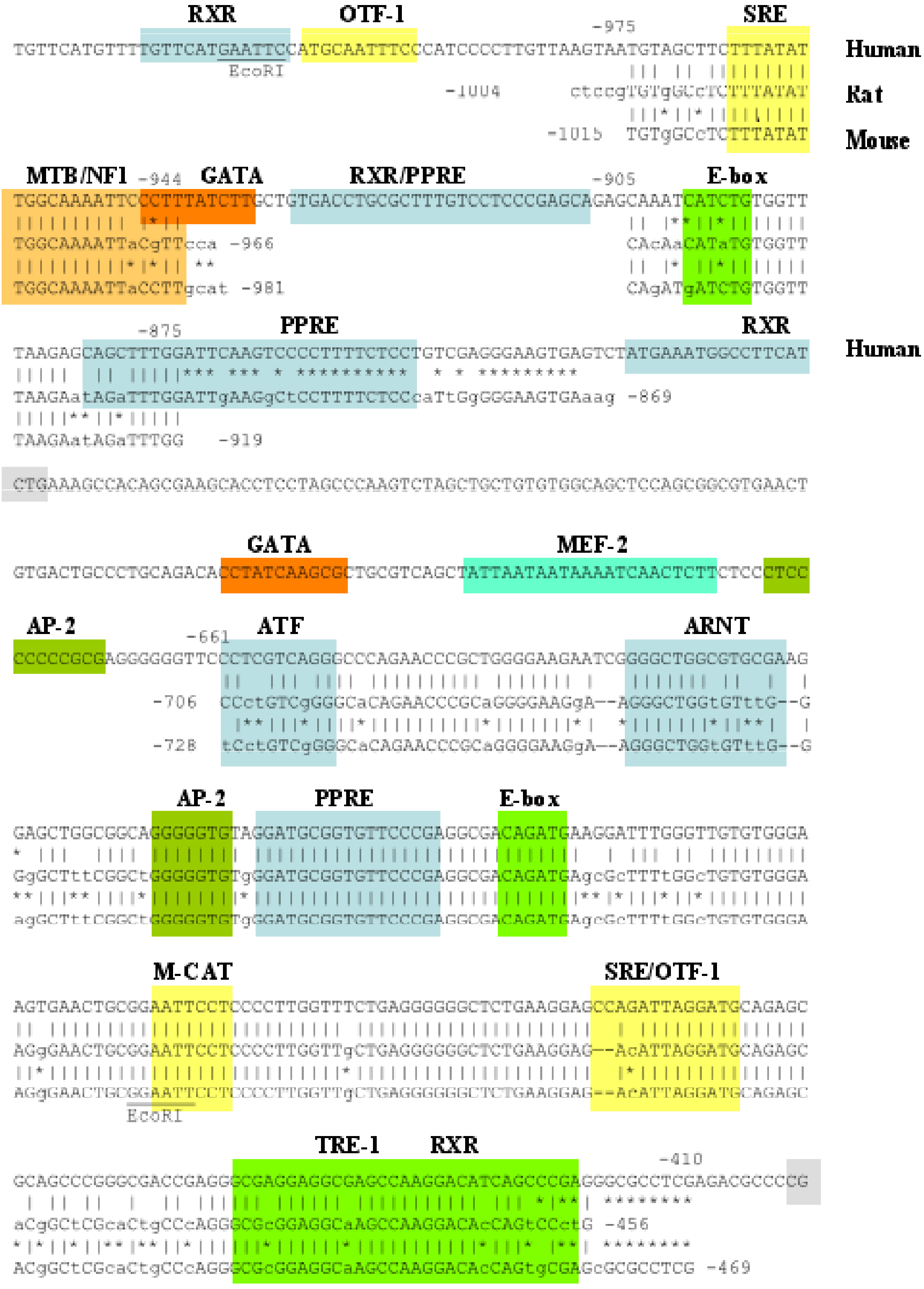

FIGURE 4., cont.

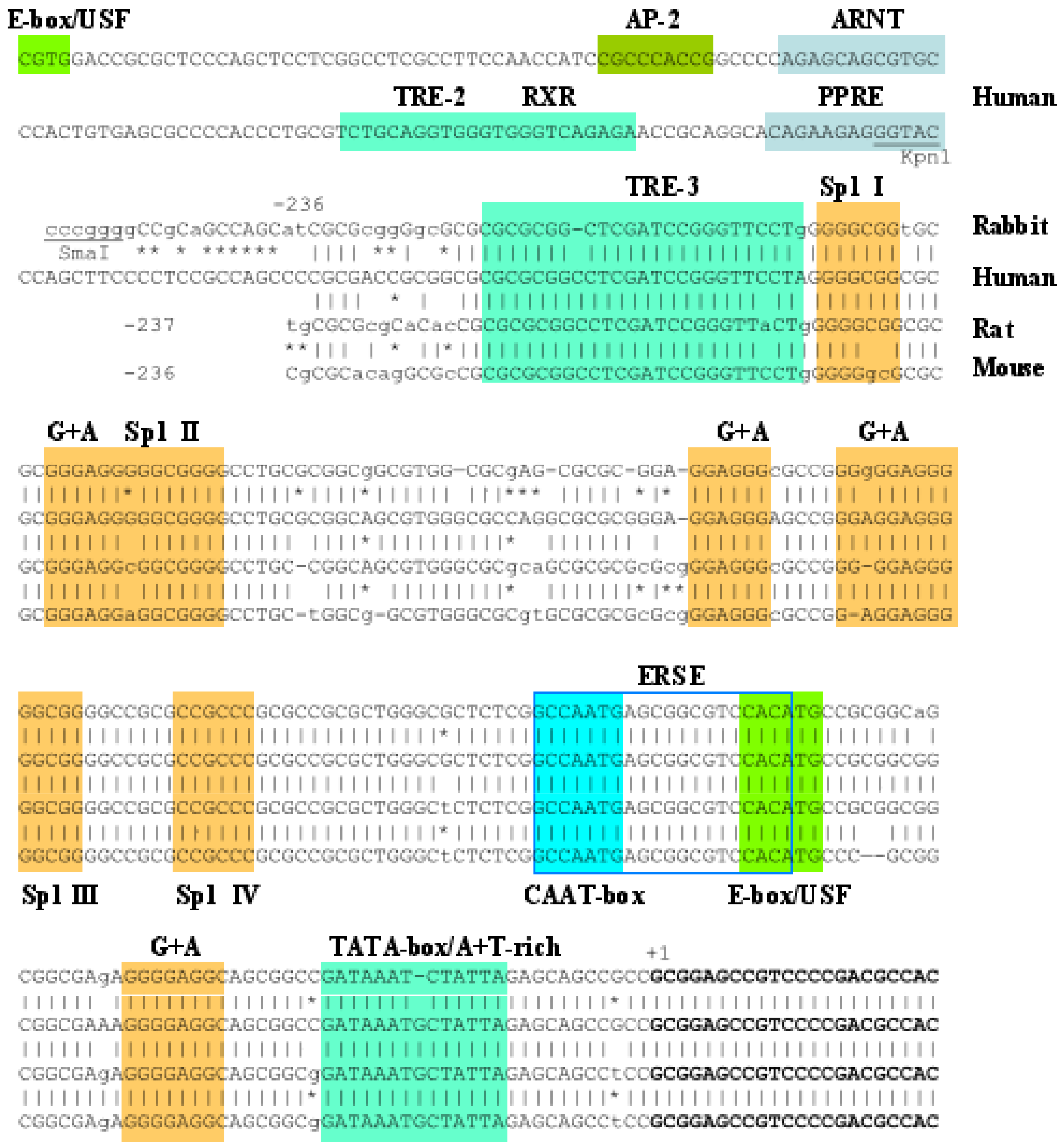

**FIGURE 4**, cont.

homology among all the SERCA2 genes cloned. In addition, in the human gene, several families of repeated sequences (L2, Alu, CAAAA, MER, MIR, LIMB, etc.) are located upstream from position -1.7 kb interspersed in a continuous manner for more than 40 kb. The third important observation is that the rabbit 5’-flanking sequence only shares homology with the mouse, rat, and human sequences in the first and third regions, but there is no homology between positions -250 and -1170 bp of the rabbit sequence[6]. Therefore, one could speculate that the rabbit SERCA2 regulatory region could be modulated in different manner.

## Functional Studies of SERCA2-Promoter-Luciferase Constructs

In order to analyze DNA regions which regulate the transcriptional activity of the human SERCA2 gene in cardiac myocytes, constructs containing -2529, -1741, -1232, and -259 bp of 5'-flanking region were transiently transfected into neonatal rat cardiomyocytes (Fig. 5A). The shortest construct containing the proximal conserved promoter region showed the maximal activity, three- to fivefold higher than constructs containing at least -1.2 kb of regulatory region (Fig. 5B). These results indicate that the proximal regulatory region is necessary and sufficient for cardiac-muscle-specific expression. It is also apparent that the region located between -225 and -1232 bp contains regulatory elements such as GATA, SRE, and M-CAT which modulate the expression of the gene in negative fashion. There was no significant difference in transcriptional activity among constructs containing -1.2, -1.7, and -2.5 kb of 5'-flanking sequence.

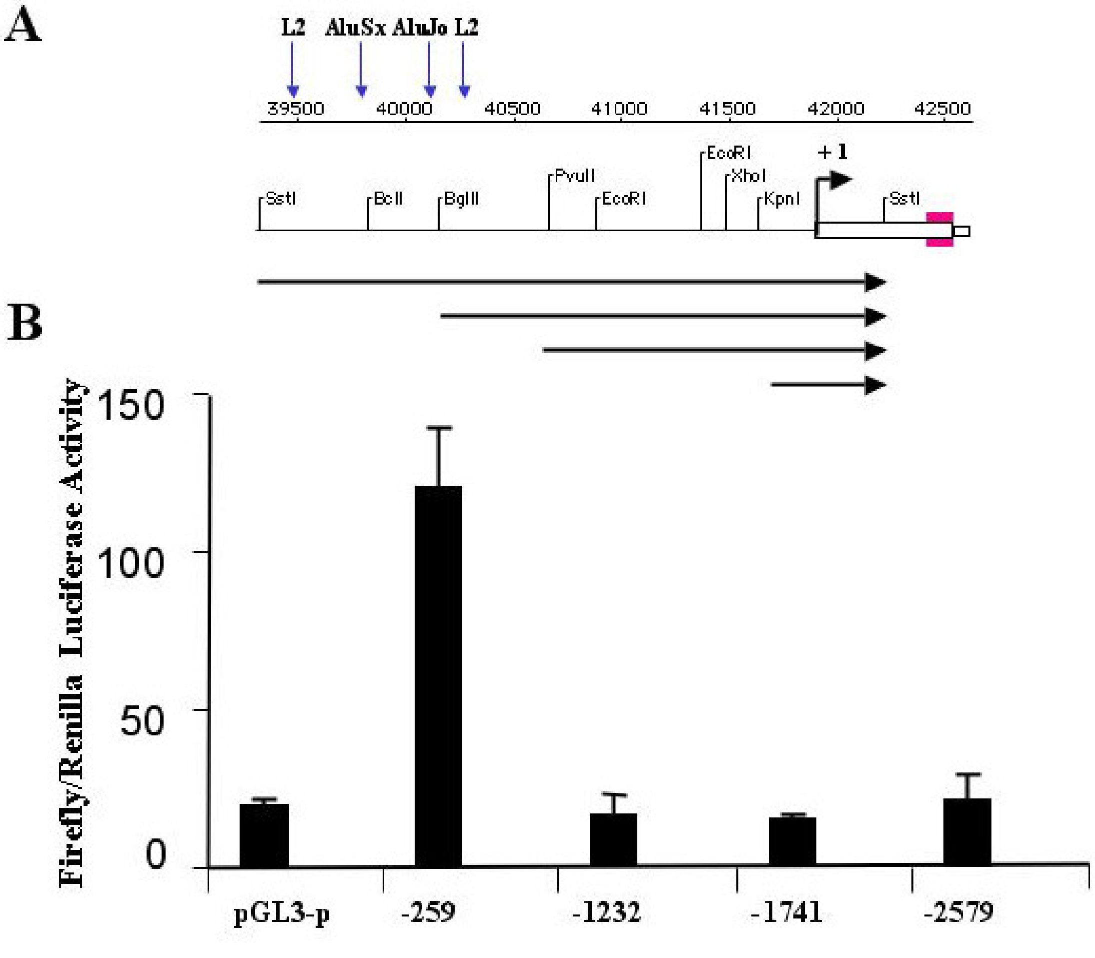

**FIGURE 5.** Design and transcriptional activity of hSERCA2 5'-regulatory region/luciferase constructs. (A) Restriction mapping of the 5'-regultory region of the human SERCA2 gene is indicated. Deletion constructs containing -259, -1,232, -1,741, and –2,579 bp are indicated with arrows. The transcription initiation site is indicated with (+1). The positions of the repeat sequences L2 and Alu are shown with arrows on top of the bar indicating the nucleotide position in the genomic clone RPC 13-305I20. (B) Neonatal rat cardiac myocytes were transfected with SERCA2/Luc constructs using Lipofectamine as described under Methods. The transcriptional activity of the hSERCA2/Luciferase constructs is shown as the normalized ratio of Firefly/Renilla luciferase activities (mean+/-S.D.). The pGL3-promoter plasmid was used to compare relative transcriptional strength.

Functional studies have shown that the proximal promoter region of the SERCA2 gene is sufficient to regulate the expression of the SERCA2 gene during myogenic differentiation of the mouse skeletal cell lines $C_2C_{12}$ and Sol8[6,16]. This region also confers high transcriptional activity in cardiomyocytes[10]. Although the proximal promoter region is necessary and sufficient for muscle-specific expression, other distal sequences contained within 1.5 kb of regulatory region are probably important for regulated expression of the gene during cardiac hypertrophy. Among the DNA cis-elements present in the distal promoter region are potential binding sites for: GATA-4, -5, -6, Nkx-2.5/Csx, octamer factor-1 (OTF-1), upstream stimulatory factor (USF), myogenic enhancer factor-2 (MEF-2), serum response factor (SRF), PPAR/RXR, AP-2, and thyroid hormone receptors, as well as E-box and M-CAT sequences. In Sol8 cells, an E-box/A+T-rich element located in the distal 5'-regulatory region of the rabbit SERCA2 gene has been suggested to contribute to the muscle-specific expression of this gene[17]. Therefore, it appears that the entire functional 5'-regulatory region of the SERCA2 gene is most likely confined within the 1.5 kb upstream from the transcription initiation site. The functional analysis presented in this study is in agreement with previous reports and suggest that regulatory elements necessary for SERCA2 expression in cardiac muscle are located within 1.2 kb of 5'-flanking region.

## SERCA2 Transcription is Modulated by Thyroid Hormone and Stress Agents

The SERCA2 gene expression is up-regulated by thyroid hormones[7,10,11,12,18]. Three TREs have been described in the rat SERCA2 gene. TRE-1 is located upstream from the proximal promoter in second conserved region of homology (-481 to -456 bp), TRE-2 is located in the region that does not share high homology (-310 to -287 bp), whereas TRE-3 is located within the proximal 225 bp of promoter sequence (-219 to -194 bp) (Fig. 4)[11,12]. TRE-1 and TRE-3 are also present in the mouse, rabbit, and human genes[8,9,10]. TRE-1 is a direct repeat of two half sites separated by four nucleotides that can be contacted preferably by the monomeric form of TR-α1, homodimers of TR-β, or by heterodimers TR-α/RXR. In addition, retinoic acid can also stimulate the SERCA2 gene expression by a mechanism that does not involve the complete TRE-1 but only the 5'-half site. In contrast, TRE-2 and TRE-3 are inverted palindromes of two half sites separated by 4 or 6 nucleotides which can bind homodimers of TR-α1 or TR-β[10,11,12]. Further, it has been shown for rat and rabbit SERCA2 promoter/reporter fusion gene constructs containing the proximal 500 bp of regulatory region that can be transactivated by TR isoforms α-1 and β-1[10,11,12]. Using the rat heart–derived cell line H9c2, it has been demonstrated that the transcription factor MEF2a increases the transcription of the rat SERCA2 gene induced by the TR-α1 and TR-β1[12]. Recently, it was reported that TR-α1, TR-α2, and TR-β1 follow different changes in the pattern of expression in physiological vs. pathological cardiac hypertrophy[19]. The three TR subtypes are down-regulated in pressure overload animal models and in cultured cardiomyocytes treated with phenylephrine[19]. In contrast, TR-β1 was up-regulated in cardiomyocytes in culture treated with triiodothyronine or in exercise-induced cardiac hypertrophy[19]. These findings explain, at least in part, why a decreased SERCA2 expression is observed in certain cardiac hypertrophy models in spite of normal circulating levels of thyroid hormones.

SERCA2 expression can be induced by endoplasmic reticulum (ER) stress agents such as intracellular $Ca^{2+}$ depletion or by agents that affect glycosylation of proteins like tunicamycin, dithiothreitol, or L-azetidine-2-carboxylic acid[20,21,22]. A recent study has shown that the proximal SERCA2 promoter region contains a functional consensus endoplasmic reticulum stress responsive elements (ERSE) that is active in response to $Ca^{2+}$ depletion[22]. The ERSE present in the SERCA2 promoter (CCAATN9CCACA) could bind NF-Y/CBF, YY-1, and ERSF (Fig. 4). The same study demonstrated that ATF6 transactivates the expression of the rat SERCA2 gene, and it has been proposed that ATF6 could interact with NF-Y and also make contact with the 5'-CCACA-3' sequence of the ERSE, although this sequence differs from the consensus sequence 5'-CCACG-3'. The 5'-CCACA-3' sequence is part of the proximal E-box/USF element and may play an important role in the basal and regulated expression of this gene. It is interesting to speculate that ATF6 may not bind directly to that sequence but rather interact with another factor(s) that actually binds to the E-box/USF sequence (5'-CACATG-3') that is part of the ERSE. Finally, it is not known if the ERSE present in the SERCA2 gene responds to other stress agents in addition to calcium depletion, including increased or decreased transcription of the gene induced by changes in glucose concentration.

## SERCA2 Expression is Down-Regulated in Cardiac Hypertrophy and Failure

Many research groups have documented that, in animal models of cardiac hypertrophy and patients with heart failure, the mRNA expression of SERCA2 is decreased[18,23,24]. Recently, it has been demonstrated that the decreased SERCA2 mRNA levels arise from a reduced gene transcription[25,26,27]. The Sp1-factor mRNA levels as well as the Sp1-factor binding activity has been shown increased in pressure overloaded (PO) hearts[28,29]. Sp1 has been reported to be necessary for induction of the skeletal α-actin in pressure overload cardiac hypertrophy[28]. Recently, it was reported that two proximal Sp1 binding sites within the SERCA2 promoter (Sp1 I and Sp1 III) are responsible for the Sp1 mediated transcriptional inhibition observed in pressure overload cardiac hypertrophy (Fig. 4)[29]. Recent evidence also suggests that activation of the p38-MAPK pathway may participate in the down-regulation of SERCA2 gene transcription in response to a hypertrophic growth of the heart[30]. It can, therefore, be suggested that a decreased SERCA2 expression leads to a dysregulation of cardiac myoplasmic free [$Ca^{2+}$], and ultimately to activation of NF-AT pathway of cardiac hypertrophy. NF-AT3 when dephosphorylated is able to interact with GATA-4 binding to its consensus sequence, thereby affecting the transcriptional activity of target genes. Thus, there is a possibility that GATA binding sites in the SERCA2 promoter are involved in down-regulating SERCA2 expression during cardiac hypertrophy.

It has been reported that, in doxorubicin (DXR)-induced cardiomyopathy, SR $Ca^{2+}$ transport is decreased, as well as the SERCA2 mRNA levels and gene transcriptional activity[31,32]. DXR-induced cardiomyopathy can be prevented by a specific inhibitor of p44/42 MAPK. ANF, β-myosin heavy chain, Egr-1, and TAFII250 expression were increased by DXR treatment[31,32]. Egr-1 is a nuclear phosphoprotein with three zinc fingers that bind to the GC-rich element (CGCCCCCGC) and can modulate transcription through repressive and activating domains that involve also competition with Sp1[31]. A G+C-rich sequence is present in the Egr-1 gene and the proximal promoter of the SERCA2 genes (Fig. 4). Transfection assays located the DXR responsive element within the proximal SERCA2 promoter region, and overexpression of Egr-1 decreased the transcriptional activity of the SERCA2 gene[31]. These findings suggested that reactive oxygen species mediate their transcriptional effect on the SERCA2 gene *via* p44/42 MAPK and Egr-1. To date, other transcriptional mechanisms influencing the SERCA2 gene in pathologic conditions are incompletely understood.

## EXPERIMENTAL METHODS AND PROCEDURES

Restriction endonucleases, modifying enzymes, Lipofectamine, tissue culture reagents, and fetal bovine serum (FBS) were from Invitrogen. The human PAC clone RPC 13-305I20 was purchased from Children's Hospital Oakland BACPAC resources, Oakland, CA. The pGL3-promoter, pGL3-basic, pRL-CMV, and Dual Luciferase assay system were purchased from Promega Co. All other chemical and reagents were Molecular Biology-grade or the highest purity available.

## Neonatal Rat Cardiomyocyte Cultures

Rat cardiac myocytes (1 or 2 days old) were isolated by a modification of the procedure previously described[10]. Briefly, the rat hearts were surgically removed, ventricles were dissected, minced in 1 X ADS buffer [final concentration: 116 mM NaCl, 20 mM Hepes (pH 7.4), 1 mM $NaH_2PO_4$, 5.5 mM Glucose, 5.4 mM KCl, 0.8 mM $MgSO_4$)], and washed to remove erythrocytes, followed by dissociation with 0.3% collagenase type I (Invitrogen), 0.6% pancreatin from porcine pancreas (Sigma), and DNAase I, 1 µg/ml (Sigma). Enzymatic activities were stopped by adding cold DMEM (glutamine free and glucose 4.5 g/l) containing 100 U/ml of penicillin; streptomycin, 110 µg/ml; amphotericin B, 0.25 µg/ml; nystatin, 10 U/ml; and supplemented with 50% FBS. Cells were pelleted by low-speed centrifugation and resuspended in DMEM plus 10% FBS. The dissociated cells were pooled and plated in 12-well culture dishes coated with 1% gelatin at a density of $1–3 \times 10^5$ cells per well in DMEM supplemented with 10% FBS and antibiotic/antimycotic cocktail. Cultures were maintained at 37ºC in a humidified incubator under atmosphere of 5% $CO_2$ and 95% air during 48 h before transfection.

## Cloning of the Human SERCA2 Promoter

The DNA from the PAC clone RPC 13-305I20 containing the entire human SERCA2 gene was digested with Sst1 and separated in 1% agarose gel. The 2.9-kb Sst1 fragment contained 2,579 bp of promoter region and 323 bp of 5'-nontranslated sequence of exon 1. The fragment was isolated and purified using Geneclean II kit, and subcloned into the unique Sst1 site located in polylinker of the promoterless pGL3-basic. The orientation of the 2.9-kb Sst1 insert was confirmed by restriction enzyme analysis. Three deletion constructs were generated from the sense Sst1-pGL3 construct using the restriction enzymes BglII, PvuII, and KpnI. The resulting deletion constructs contained 1,741, 1,232, and 259 bp of 5'-flanking sequence, respectively. Plasmid constructs were amplified and purified using the Qiagen kit and protocol, and then used for DNA transfection experiments as described below.

## Transient DNA Transfections and Luciferase Assays

Plasmid constructs were transfected into primary cardiomyocytes cultures following a modification of the Lipofectamine procedure (Invitrogen). Briefly, 1 µg of the purified recombinant chimeric hSERCA2/Luc plasmids was mixed with 0.2 µg of pRL-CMV. After 4 h of incubation at 37ºC, 5% $CO_2$ the transfected cells were supplemented with DMEM containing 5% FCS and incubated at 37ºC, 5% $CO_2$ for 60 h. Transfected cells were harvested in 1X PLB (Promega), incubated for 1 h at RT under agitation, frozen at -80ºC, and thawed at RT. Cell extracts were prepared by centrifugation at 11,000 X g, for 2 min. The supernatant was collected and used to measure the Firefly and Renilla luciferase enzymatic activities using the Dual Luciferase assay system from Promega and the Victor-2 multi-well plate luminometer (Wallac). Protein was measured using the micro-BCA kit (Sigma).

## DNA Sequence Analysis

The sequence from the genomic insert in the clone RPC 13-305I20 is in Genebank with accession number AC006088. Identification of exon/intron boundaries, transcription regulatory sites ,and homology with other sequences were performed using MacVector 6.5.3 (Oxford Molecular), BLAST (NCBI), and GEMS (Genomatix) analysis packages.

## ACKNOWLEDGMENTS

This work was supported by grants from the Dirección General del Personal Académico, UNAM, and from CONACyT, México.
